# A Validated Method for the Simultaneous Measurement of Tryptophan, Kynurenine, Phenylalanine, and Tyrosine by High-Performance Liquid Chromatography–Ultraviolet/Fluorescence Detection in Human Plasma and Serum

**DOI:** 10.1021/acsomega.5c07457

**Published:** 2026-01-16

**Authors:** Lucia Parráková, Cornelia A. Karg, Stefanie Hofer, Pablo Monfort-Lanzas, Celina Wilgermein, Kevin Allmer, Sabine Scholl-Bürgi, Anita Siller, Harald Schennach, Simon Geisler, Dietmar Fuchs, Thomas K. Felder, Johanna M. Gostner

**Affiliations:** 1 Institute of Medical Biochemistry, Biocenter, 27280 Medical University of Innsbruck, Innsbruck 6020, Austria; 2 Institute of Bioinformatics, Biocenter, Medical University of Innsbruck,Innsbruck 6020, Austria; 3 Division of Psychiatry I, Department of Psychiatry, Psychotherapy, Psychosomatics and Medical Psychology, Medical University of Innsbruck, Innsbruck 6020, Austria; 4 Department of Laboratory Medicine, 31507 Paracelsus Medical University, Salzburg 5020, Austria; 5 Department of Paediatrics I, Medical University of Innsbruck, Innsbruck 6020, Austria; 6 Central Institute for Blood Transfusion and Immunology, University Hospital Innsbruck, Tirol Kliniken GmbH, Innsbruck 6020, Austria; 7 Institute of Biological Chemistry, Medical University of Innsbruck, Innsbruck 6020, Austria; 8 Institute of Pharmacy, Paracelsus Medical University, Salzburg 5020, Austria; 9 Core Facility Metabolomics II, Biocenter, Medical University of Innsbruck, Innsbruck 6020, Austria

## Abstract

Aromatic amino acids are precursors of neurotransmitters and immunomodulatory molecules, and their catabolism is dysregulated in various disorders associated with inflammation. This dysregulation often correlates with disease stage, symptom severity, comorbidities, quality of life, and cognitive performance, making its measurement valuable for research, diagnostics, and personalized monitoring. We developed a rapid, reliable, and cost-effective HPLC method for the simultaneous quantification of phenylalanine, tyrosine, tryptophan, and kynurenine in human serum and plasma. After protein precipitation, analytes were separated on a reversed-phase C18 column under isocratic conditions. Detection was performed based on intrinsic fluorescence for tryptophan, phenylalanine, and tyrosine and on UV absorption for kynurenine and the internal standard nitrotyrosine. The method showed linearity (R^2^ > 0.99) over 0.31–20 μM for kynurenine, 1.56–200 μM for phenylalanine, 0.08–200 μM for tryptophan, and 0.78–200 μM tyrosine. Limits of detection were 0.01 μM for tryptophan, 0.08 μM for tyrosine and kynurenine, and 0.39 μM for phenylalanine. Precision and accuracy were within 15%, and recovery rates ranged from 98 to 100%. Samples remained stable after processing and after three freeze–thaw cycles. Interlaboratory testing confirmed the reproducibility of the results. This validated method enables sensitive, accurate, and simultaneous quantification of key aromatic amino acids, providing a practical alternative to LC–MS/MS for routine diagnostics and biomarker studies.

## Graphical abstract

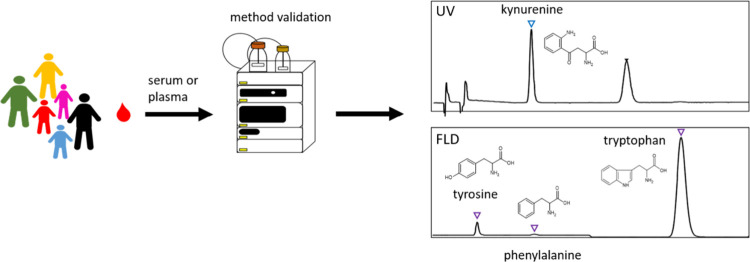

## Introduction

Aromatic amino acids (AAs) such as phenylalanine (Phe), tryptophan (Trp), and tyrosine (Tyr) are not only essential building blocks of proteins but also serve as precursors to numerous bioactive molecules involved in neurotransmission, immune regulation, and redox homeostasis. Dysregulation of their metabolism has been linked to a wide range of pathological conditions, including infections, autoimmune diseases, neurodegenerative disorders, malignancies, and psychiatric disorders.^1–7^ Reliable determination of these AAs and their ratios in biological fluids is therefore of considerable interest for research, diagnostics, and personalized medicine.

The Trp-kynurenine (Kyn) pathway is particularly relevant in this context. Trp is the precursor for several biosynthetic pathways, including the formation of the neurotransmitter serotonin, which plays a key role in mood regulation and various physiological functions.^8–10^ The Trp-Kyn pathway metabolizes more than 95% of Trp in the human body, resulting in the formation of kynurenic acid, xanthurenic acid, and nicotinamide.^11^ The extrahepatic indoleamine 2,3-dioxygenases (IDO) 1 and 2, as well as hepatic tryptophan 2,3-dioxygenase, are able to catalyze the first and rate-limiting step in this pathway, the formation of N-formylkynurenine, which is then hydrolyzed to the more stable Kyn. Kyn can then be further metabolized to either kynurenic acid, anthranilic acid, or 3-hydroxykynurenine (Figure 1A). IDO-1 is induced by proinflammatory cytokines, most importantly interferon-γ, mainly in monocyte-derived cells, but also in other immune and nonimmune cells. IDO-1 induction in peripheral blood cells causes decreased Trp and increased Kyn levels and is often considered as an indicator of cellular immune activation.^12^ Dysregulated Trp catabolism and changes in Kyn downstream metabolite levels in the serum and plasma of patients have been reported in viral infections, neurodegenerative disorders, various malignancies, metabolic and neuropsychiatric disorders.^5,13–15^ Already in the early 1990s, the Kyn to Trp ratio (Kyn/Trp) was proposed as an estimate of IDO enzyme activity when accompanied by an elevation of other immune activation markers such as neopterin.^13^

**Figure 1:**
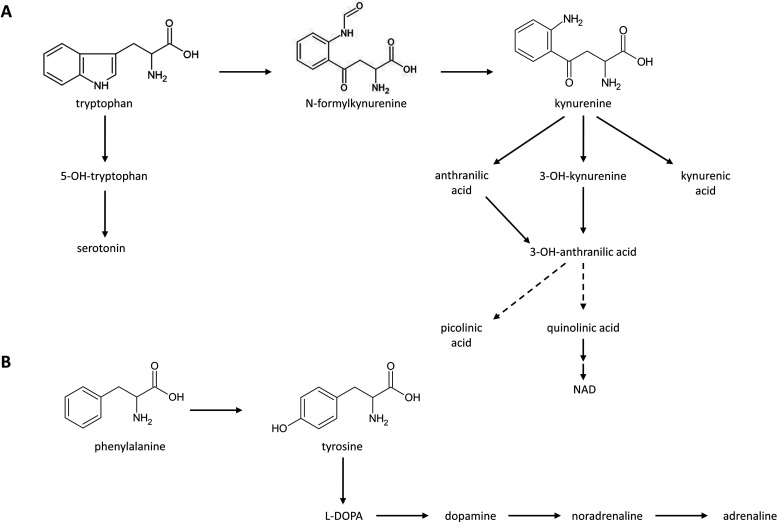
Metabolic pathways of Trp (A) and Phe (B). (A) Trp is primarily catabolized through the Kyn pathway. N-formylkynurenine is converted to the more stable Kyn. Kyn can subsequently be metabolized to kynurenic acid, anthranilic acid, or 3-hydroxykynurenine. In addition, Trp serves as a precursor for serotonin. (B) Phe is converted to Tyr, which is further hydroxylated to L-DOPA.

The Phe-Tyr pathway provides a complementary perspective on inflammation-associated changes in AA metabolism. Reduced Phe turnover is a nonspecific indicator of inflammation and oxidative stress,^16^ and dysregulated metabomism has been reported in trauma, sepsis, cancer, and treatment-naïve HIV patients.^4,17–19^ The conversion of Phe to Tyr is catalyzed by Phe hydroxylase (PAH), which requires the cofactor tetrahydrobiopterin (BH_4_) (Figure 1B). BH_4_ is also needed for the biosynthesis of the catecholamine precursor L-3,4-dihydroxyphenylalanine (L-DOPA) from Tyr, and for serotonin synthesis. The tetrahydropteridine derivative BH_4_ is chemically labile and prone to irreversible oxidation.^20^ Loss of BH_4_ in chronic inflammatory conditions may impair neurotransmitter synthesis.^4^ Inflammation-associated changes in neurotransmitter precursor metabolite profiles could contribute to the development of neuropsychiatric symptoms.^21^ Preanalytics and measurements of BH_4_ itself are very demanding, thus the Phe to Tyr ratio (Phe/Tyr) is frequently applied as a surrogate marker of PAH activity.^16,22^

A variety of analytical methods exist for detecting AAs using capillary electrophoresis, gas chromatography, or high-performance liquid chromatography (HPLC), coupled with ultraviolet (UV), electrochemical, fluorescence, or mass spectrometry detection.^23,24^ Conventionally, AA serum concentrations have been measured by HPLC and ninhydrin derivatization followed by photometry, though this method can be time-consuming.^23^ However, liquid chromatography tandem mass spectrometry (LC-MS/MS) has become a favored technique due to its high specificity and shorter analysis time. Nevertheless, weak ionization of AAs and the possibility of matrix effects may result in loss of precision. MS techniques generally require costly equipment and specialized laboratories, and limits of quantification are often not superior than those achieved by conventional techniques.^25^ Derivatization can overcome weak ionization but may increase variability of results as reported from interlaboratory studies.^26–28^ Relevant factors are unstable derivatives, reagent interference, incomplete derivatization, side reactions, long analysis times and additional peaks in chromatograms.^27,28^ These complexities are further amplified when working with crude extracts, highlighting the need for simple sample preparation, short analysis times, and high sensitivity to increase the reliability of AA analysis.^27^

Based on the combination of two well-established classical methods,^29–31^ we present a fast and accurate HPLC-UV/FLD method for the simultaneous analysis of Trp, Phe, Tyr and Kyn in human serum and plasma, without the need of derivatization. The natural fluorescence of Trp, Phe, and Tyr, and the UV absorption of Kyn are used for detection. This method provides a cost-effective and straightforward alternative to device and sample preparation-intensive methods, thus being ideally suited to be implemented in research and clinical laboratories.

## Materials and Methods

### Chemicals

The standard compounds l-tryptophan (l-Trp), L-kynurenine (L-Kyn), l-tyrosine hydrochloride (l-Tyr HCl), l-phenylalanine (l-Phe), and nitrotyrosine (NT, internal standard (IS)) were obtained from Sigma-Aldrich, Vienna, Austria. All other chemicals were obtained from Sigma-Aldrich, Vienna, Austria, if not otherwise stated. Albumin was obtained from Serva Electrophoresis GmbH, Heidelberg, Germany. Trichloroacetic acid was purchased from Carl Roth, Karlsruhe, Germany. Deionized water was prepared using a purification system from Sartorius, Göttingen, Germany.

### Serum and Plasma

Anonymized residual blood from blood donors of the *Central Institute for Blood Transfusion* and *Immunology of the University Hospital Innsbruck, Austria*, who consented to the use of residual blood for scientific purposes, was used for the preparation of the matrix and for the comparative analysis.

#### Plasma for Matrix Preparation

Whole blood from blood donations was drawn into bags containing citrate-phosphate-dextrose as anticoagulant and stored overnight below 24 °C. Plasma was prepared by centrifugation of whole blood at 1730 g for 10 min at 4 °C and then stored at – 20 °C. Only whole blood from aborted blood donations was used, which could not be used for transfusion.

#### Serum for Matrix Preparation and Interlaboratory Comparison

Whole blood from donors was drawn in Vacuette CAT Serum Clot Activator Tubes. The blood samples were cooled at 4 °C and stored in the dark. The next day, blood samples were centrifuged at 3916 g for 10 min at 10 °C before routine infection serology was performed. From each donor, approximately 500 μL of serum was collected after the samples had passed through the analysis line. 80 serum samples were pooled in order to have sufficient serum for matrix preparation.

### Preparation of Matrices

Charcoal-depleted plasma (CDP) and serum (CDS) were prepared by adding 1.68 g of activated charcoal to 30 mL of plasma and to 30 mL of serum. Samples were mixed on a rotary incubator for 5 h at room temperature or, alternatively, overnight at 4 °C. Plasma/serum was centrifuged at 5125 g for 15 min at 4 °C and the supernatant was filtered through a sterile filter (0.2 μm). Depleted plasma/serum was aliquoted and stored at −20 °C. A 7% (w/v) solution of human serum albumin was prepared as an alternative matrix to match the total protein of human serum and plasma by dissolving 35 g of albumin and 4.5 g of NaCl in 500 mL of deionized water.

### Sample Preparation

Stock solutions of AAs were prepared in albumin and in deionized water, aliquoted and stored at −20 °C until further use. Calibrator mix solutions were prepared freshly before every measurement by adding equal volumes of freshly thawed stock solutions of l-Trp, L-Kyn, l-Tyr hydrochloride and l-Phe to reach a final concentration of 200 μM for Trp, Tyr, Phe, and 20 μM for Kyn.

Quality control (QC) and control samples were prepared by mixing known volumes of working stocks with albumin, CDP, or CDS to obtain two different concentrations at low or high levels, respectively, related to the calibration range (Table 1 or as indicated). QC and control samples were stored at −20 °C until analysis, but for at least 24 h.

**Table 1:** Concentrations of Quality Control (QC) Samples^a^

	QC low [μM]	QC high [μM]
Kyn	1.5	15
Phe	15	150
Trp	15	150
Tyr	15	150

a The concentrations were selected according to their occurrence in human blood samples (lower and higher concentration ranges).

NT was used as an IS and was prepared by diluting a working stock of 500 μM to a final concentration of 25 μM in deionized water. 100 μL of each calibrator mix solution, quality control samples, or real samples were mixed with 100 μL of IS. Proteins were precipitated by adding 25 μL of 2 M trichloroacetic acid. Tubes were vortexed immediately and centrifuged for 10 min at 16060 g at room temperature. The supernatant was transferred to a new tube followed by an additional centrifugation step for 10 min at 16060 g. The supernatant was transferred into HPLC vials for analysis.

### HPLC-Method Based on RP-18 and UV/FLD Detection

The method validation was performed on an Agilent 1260 Infinity II LC system with a 1260 Infinity Degasser, a 1260 Series quaternary pump, 1260 auto sampler, 1260 column thermostat, 1260 Series diode array detector (DAD) and a 1260 fluorescence detector (Reference Laboratory, Instrument 1). For assessing laboratory precision, samples were measured on an Agilent 1100 system equipped with a 1100 degasser, a 1100 Series quaternary pump, 1100 Series diode array detector and 1100 fluorescence detector (Reference Laboratory, Instrument 2).

AAs were analyzed on a Merck Purospher STAR RP-18 (3 μm) LiChroCART 55–4 column, protected by a Phenomenex100 RP-18 (5 μm) LiChroCART 4–4 i.d. precolumn. Analytes were eluted after injecting 30 μL of sample volume using an isocratic elution with potassium dihydrogen phosphate (KH_2_PO_4_), 15 mM, pH 4.6 as mobile phase and a flow rate of 1.1 mL/min for 10 min at 25 °C. Kyn and NT were detected at a wavelength of 360 nm. Tyr and Phe were detected by fluorescence detection at an excitation wavelength of 210 nm and an emission wavelength of 302 nm (1–5 min); Trp at 286/366 nm (ex/em) (5–10 min). UV–vis and fluorescence spectra of Trp, Tyr, Kyn and NT can be found in Supplementary File 1 (Figure S1 and S2). NT was used as IS for both UV and fluorescence traces. Control measurements showing no additional peaks in the fluorescence chromatogram confirmed that Kyn and NT were not detectable at the excitation/emission wavelengths applied for Trp, Phe, and Tyr. Kyn shows only weak fluorescence at 365/480 nm, and NT is essentially nonfluorescent due to quenching by its nitro group on the aromatic ring^32,33^ (see Supplementary File 1, Figure S2). Data was collected and processed with OpenLab CDS Data Analysis 3.4.

Interlaboratory assessment was performed (i) with the identical method, for which columns, solvents, calibrators, and QCs were provided by the leading laboratory and measured on a Shimadzu LC-40 HPLC. The system included a controller CBM-40 CL, a degassing unit DGU-405 CL, a solvent delivery module LC-40D XR CL, an autosampler SIL-40C XR CL, a column oven CTO-40C CL, a UV–vis detector SPD-40 CL and was controlled by LabSolutions CL v1.40 software (Shimadzu Corporation, Kyoto, Japan) (External laboratory 1). (ii) In addition, an HPLC method based on ion-exchange resin and ninhydrin-derivatization was used (External laboratory 2), for which serum samples were prepared and analyzed using an automated AA analyzer (Biochrom 30+, Biochrom, Cambridge, UK) as reported previously.^24,34^

### Method Validation Protocol

Method validation was performed using the validation guidelines of the *Gesellschaft für Toxikologische and Forensische Chemie (GTFCH)*.^35^ The method was validated for selectivity, linearity, intra- and interday precision, accuracy in either albumin solution (used as a validation matrix), CDP, or CDS, respectively, and stability and recovery in albumin.

#### Selectivity

The method’s selectivity was verified by analyzing albumin blanks from three different charges, as well as plasma and serum from three different healthy donors. Six blanks without IS as well as albumin, plasma, and serum blanks containing IS were measured. These results were then compared with blank matrices containing a standard mixture of all four analytes, including the IS (Supplementary File 1, Figure S3).

#### Calibration

The calibration range was determined to cover the expected concentration levels of the analytes in authentic samples. To achieve this, calibrators were prepared by spiking three different blank matrix samples (albumin, CDP, and CDS) with different concentration levels of the analytes, ensuring that the lowest concentration was equal to the lower limit of quantification (LLOQ) and the highest concentration was within the upper limit of quantification (ULOQ). Peak area ratios (analyte/IS) were plotted against the different concentrations of the calibration range and a linear regression was used for each analyte in all matrices. The linear regression analysis of eight independent measurements yielded equations and the coefficient of determination (R^2^) for every analyte in all matrices (Table 2). Outlier detection was performed using Grubb’s test statistic (G). The test was performed on data obtained from eight determinations at each concentration level. The significance level was set at 95% (α < 0.05).

**Table 2:** Summary of Validation Results in Albumin, Charcoal-Depleted Plasma (CDP) and Charcoal-Depleted Serum (CDS) Including Retention Time, Calibration Range, Linear Equation, Coefficient of Determination (*R* ^2^), Limit of Detection (LOD), and Lower Limit of Quantification (LLOQ)

matrix	analyte	retention time (min)	calibration range (μM)	linear equation	***R*** ^2^	LOD (μM)	LLOQ (μM)
albumin	Kyn	3.1	0.31–20	*y* = 15.59*x* – 0.07	0.999	0.08	0.31
	Phe	3.2	1.56–200	*y* = 2644.24*x* – 0.26	0.994	0.39	1.56
	Trp	7.9	0.08–200	*y* = 8.43*x* – 0.22	0.996	0.01	0.08
	Tyr	1.5	0.78–200	*y* = 223.94*x* – 0.50	0.996	0.08	0.39
CDP	Kyn	3.1	0.63–20	*y* = 16.59*x* + 0.40	0.995	0.31	0.63
	Phe	3.2	1.56–200	*y* = 2903.90*x* + 0.60	0.997	0.39	1.56
	Trp	7.8	0.78–200	*y* = 9.17*x* + 1.92	0.998	0.19	0.78
	Tyr	1.5	0.78–200	*y* = 231.06*x* + 0.51	0.998	0.08	0.78
CDS	Kyn	3.1	0.31–20	*y* = 16.74*x* + 0.27	0.997	0.16	0.63
	Phe	3.2	6.25–200	*y* = 2909.80*x* + 0.75	0.998	0.78	6.25
	Trp	7.8	6.25–200	*y* = 9.12*x* + 3.46	0.998	0.78	6.25
	Tyr	1.5	1.56–200	*y* = 233.18*x* + 0.50	0.999	0.39	1.56

#### Limit of Detection (LOD) and Lower Limit of Quantification (LLOQ)

The limit of detection (LOD) and lower limit of quantification (LLOQ) were determined based on signal-to-noise ratios (S/N) of 3 and 10, respectively. S/N-ratios were calculated using the Peak-to-Peak (P2P) method of the OpenLab software; a baseline subtraction within a fixed time region was performed for every peak.

#### Accuracy and Precision

To assess the accuracy of the method, a series of QC samples were prepared. These QC samples were designed to cover a range of concentrations, including low and high levels, compared to the calibration range. A minimum of two QC samples at each concentration level were analyzed over eight different days. This provided a reliable assessment of method precision for the analysis of the metabolites in albumin, CDP, and CDS matrices. Concentrations of AAs were calculated using the respective linear calibration in either albumin, CDP or CDS.

#### Bias

The systematic error (bias) as a percentage was calculated by comparing the average of the QC sample measurements over the 8 days with the accepted reference values.

#### Repeatability

The Repeatability Standard Deviation (RSDr) was calculated to assess the precision or repeatability of measurements within a single day.

#### Time-Different Intermediate Precision

RSD_(T)_ (Repeatability Standard Deviation for Time-different Intermediate Precision) was calculated to estimate the reliability of measurements obtained from repeated analyses of the same sample or of measurements performed over multiple days, each day involving multiple repetitions. In addition, the 95% β-tolerance interval was calculated.

Laboratory precision was determined by measuring QC samples on a different HPLC device, by preparing QC samples by a different operator, and by preparing and measuring QC samples in a different laboratory (different operator and device).

#### Stability

Processed sample stability was determined by preparing 6 control samples in albumin at low and high concentration levels each, pooling them, and preparing 6 aliquots again, which were then measured at regular intervals over a duration corresponding to the expected length of a regular analysis series.

For the determination of freeze/thaw stability, six control samples at low and high concentrations were compared with six control samples at low and high concentration levels that had been exposed to three freeze/thaw cycles.

#### Recovery and Extraction Efficiency

Recovery was determined by analyzing calibrator dilutions in albumin and in water at six different concentration levels and comparing the slopes of the regression lines. Extraction efficiency was assessed by the determination of control samples in albumin at low and high concentration levels, which were prepared by adding the analyte and the IS after precipitation, and control samples in albumin, which were prepared by adding the analyte to the matrix before precipitation, but the IS after precipitation.

### Statistics

All statistical analyses were conducted using in-house Python scripts or GraphPad Prism 10.0. Formulas used for the calculations can be found in Supplementary File 1, Figure S4.

## Results

The chromatographic method successfully separated Kyn, Tyr, Phe, Trp, and the IS NT within a run time of 10 min. Kyn and NT were detected by UV at a wavelength of 360 nm, whereas Tyr, Phe, and Trp were quantified by fluorescence detection at ex/em of 210/302 nm for Tyr and Phe, and 286/366 nm for Trp. The mobile phase consisted of 15 mM KH_2_PO_4_ and was run in isocratic mode, the flow rate was set to 1.1 mL/min. The method was tested in three different matrices, a 7% (w/v) albumin solution (as a simple surrogate matrix to match the total protein content of human serum and plasma) as well as charcoal-depleted plasma (CDP), and charcoal-depleted serum (CDS), to ensure broad applicability.

The method gave well separated peaks with good symmetry and average retention times of 3.1 min for Kyn, 6.2 min for the IS NT, 1.5 min for Tyr, 3.2 min for Phe, 7.9 min for Trp in albumin, and 7.8 min for Trp in CDS and CDP (Figure 2, Table 2).

**Figure 2:**
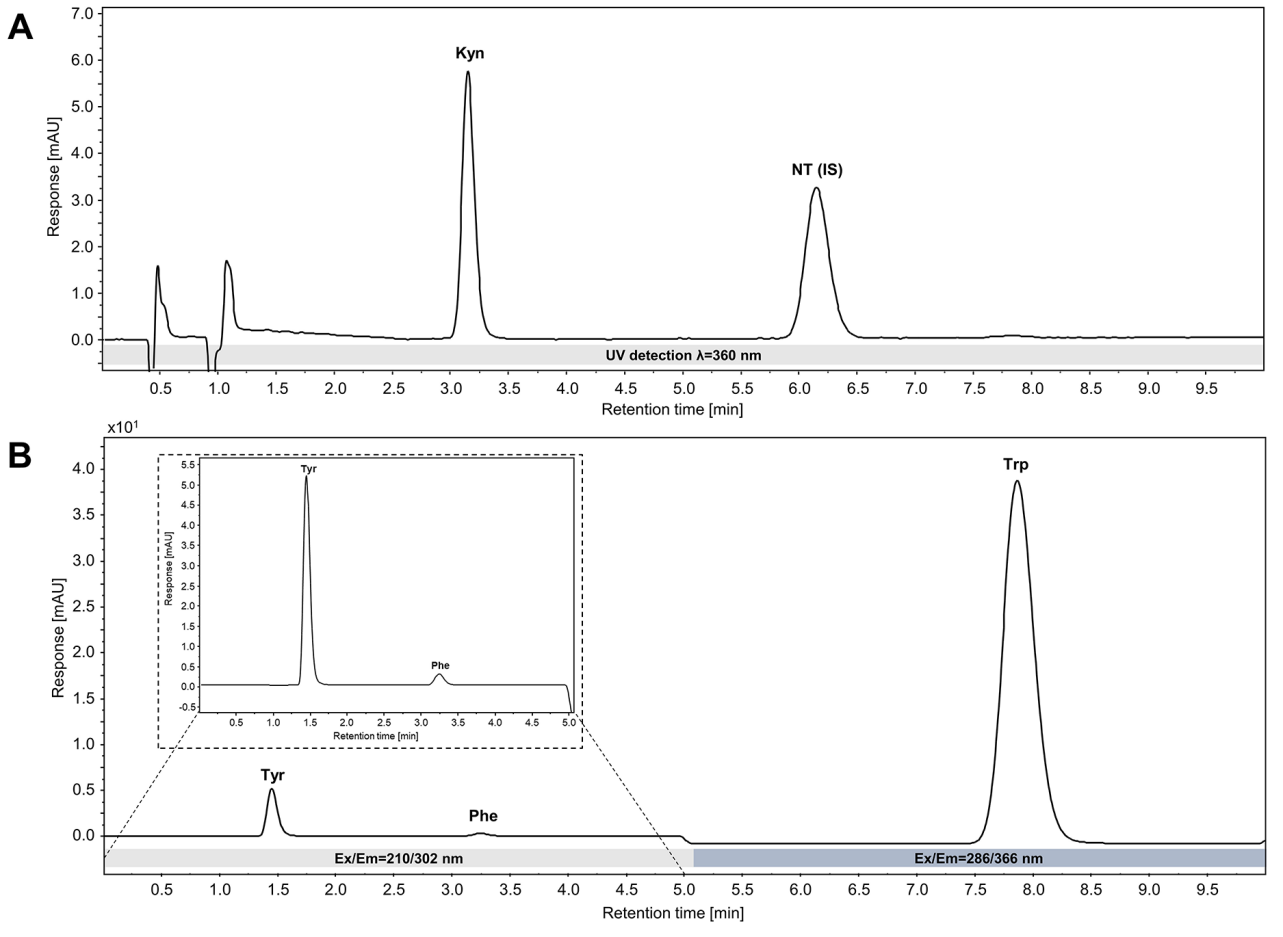
Chromatograms of a standard mix in albumin matrix (Kyn = 15 μM, Tyr/Phe/Trp = 150 μM, and NT­(IS) = 25 μM) obtained with the developed method parameters. (A) UV detection of Kyn and NT at λ = 360 nm. (B) Fluorescence detection of Tyr and Phe at λ ex/em = 210/302 nm (0–5 min; zoom) and Trp at λ ex/em = 286/366 nm (5–10 min).

The method was successfully validated according to the guidelines of the *Gesellschaft für Toxikologische and Forensische Chemie (GTFCH)* for **selectivity**, **linearity**, **intraday precision, time-different intermediate precision**, **accuracy** in either albumin solution or charcoal-depleted plasma or serum of healthy donors, respectively, and **recovery** and **stability** in albumin matrix. AAs are endogenous analytes. By charcoal-stripping, analyte-reduced plasma and serum matrices were obtained. However, a blank of all matrices was measured in addition to all measurements and for determining LOD and LLOQ values baseline subtractions of blank matrices in the range of the respective peaks were performed.

The **selectivity** of the method, the ability to detect and identify the compounds of interest without interference from other compounds, was confirmed by the absence of interfering signals from endogenous compounds or degradation products in the blank matrices at the same retention times as the analytes (Supplementary File 1, Figure S3).

The calibration was tested with water standards and compared with albumin, CDP, and CDS matrix standards and found to be equivalent. As a result, albumin, CDP, and CDS matrices were then used for analyzing the respective QC samples, and albumin was used to analyze real serum samples. Calibration curves were generated by plotting the peak area ratio (analyte/IS) against the concentrations. Linear calibrations (R^2^> 0.99/n = 8) were obtained for all analytes in a calibration range of 0.31–20 μM for Kyn, 1.56–200 μM for Phe, 0.08–200 μM for Trp, 0.78–200 μM for Tyr in albumin, 0.63–20 μM for Kyn, 1.56–200 μM for Phe, 0.78–200 μM for Trp, and 0.78–200 μM for Tyr in CDP, and 0.31–20 μM for Kyn, 6.25–200 μM for Phe, 6.25–200 μM for Trp, and 1.56–200 μM for Tyr in CDS. All concentrations of the calibration ranges were tested for outliers by a Grubbs-test with a 95% significance level and met the requirements of the guideline (no more than two outliers were allowed at each concentration level, and these outliers could not occur simultaneously). The calibration range, linear equations, coefficient of determination (R^2^), LOD, and LLOQ are summarized in Table 2. LOD and LLOQ values were evaluated using the signal-to-noise ratio and were lower than 0.8 μM for all analytes except Phe, for which a LLOQ of 1.56 μM in albumin and CDP and of 6.25 μM in CDP was obtained, and except for Trp and Tyr, for which a LLOQ of 6.25 μM and 1.56 μM was determined in CDS, respectively. These data are consistent with literature data.^29,31,36^

The **precision** and **accuracy** of the method were acquired for each analyte in all matrices. Table 3 summarizes the results of precision data, including interday and time-different intermediate precision, bias, the acceptance interval for bias and precision (95% β-tolerance interval), and the recovery and extraction efficiency in albumin. A lower RSDr and RSD_(T)_ value indicates higher repeatability and precision within a group of measurements. RSDr and RSD_(T)_ of all analytes did not exceed 10% (according to the guideline ≥ 15% is regarded as acceptable). The level of trueness of the method was expressed as bias in % and turned out to be within ± 10.8% for all analytes in all matrices. Only the low QC value of Trp in CDS exceeded 15% (16.3%), but still remained within the acceptable limit of 20% of the LOD as specified by the guidelines. In addition to bias and precision the 95% β-tolerance interval was determined and appeared to be within an acceptance interval of ± 30% for all analytes, except for the low QC value of Kyn in CDP (32.85%), which still remained within the extended limit of ± 40% of the LOD. To evaluate the analytical recovery of the proposed method, albumin calibration was compared with a water calibration of 100%. The calculated values ranged between 98 and 100%. For extraction efficiency, control samples of two different concentrations (low: 2 μM for Kyn, 20 μM for Phe, Trp, Tyr; high: 20 μM for Kyn, 200 μM for Phe, Trp, Tyr) were measured, and all obtained ratios of extracts vs control samples varied only between 93 and 104%.

**Table 3:** Summary of Validation Results in Albumin, Charcoal-Depleted Plasma (CDP), and Charcoal-Depleted Serum (CDS), Including Repeatability (RSDr) and Time-Different Intermediate Precision (RSD_(T)_), Bias, 95% β-Tolerance Interval, Recovery (R), and Extraction Efficiency (EE)^a^

Matrix	Analyte	QC concentration (μM)	RSDr (%)	RSD_(T)_ (%)	Bias (%)	95% β-tolerance interval (%)	R (%)	EE (%)
albumin	Kyn	1.5	3.5	4.1	–2.6	–12.01, 6.91	100	101
		15	1.6	3.0	–2.2	–9.35, 5.00		103
	Phe	15	2.0	3.5	–2.9	–11.26, 5.54	98	100
		150	1.5	3.0	–1.1	–8.31, 6.14		101
	Trp	15	1.9	2.7	0.1	–6.13, 6.27	98	95
		150	1.6	2.8	0.2	–6.5, 6.94		93
	Tyr	15	1.9	2.6	–2.2	–8.23, 3.76	99	104
		150	1.5	2.6	–2.4	–8.56, 3.75		101
CDP	Kyn	1.5	5.8	9.3	10.8	–11.16, 32.85		
		15	1.5	3.2	1.1	–6.59, 8.83		
	Phe	15	2.5	7.3	7.9	–9.93, 25.79		
		150	1.8	4.7	–1.9	–13.42, 9.72		
	Trp	15	1.7	4.9	4.2	–7.94, 16.30		
		150	1.6	2.9	–2.9	–9.75, 3.96		
	Tyr	15	1.6	5.0	6.2	–6.15, 18.58		
		150	1.7	3.3	–1.9	–9.89, 6.00		
CDS	Kyn	1.5	2.6	6.5	6.1	–9.82, 22.02		
		15	1.1	2.1	2.1	–2.97, 7.08		
	Phe	15	1.8	5.4	10.0	–3.38, 23.29		
		150	1.1	4.5	0.4	–10.83, 11.54		
	Trp	15	1.6	4.0	16.3	6.47, 26.15		
		150	0.9	3.0	–0.6	–8.13, 6.86		
	Tyr	15	2.1	3.8	9.2	0.07, 18.33		
		150	0.9	3.1	1.5	–6.03, 9.13		

a Results are shown for two quality control levels (QC low and QC high): upper rows correspond to QC low, lower rows to QC high. Extraction efficiency was determined for albumin at 2 μM (control low) and 20 μM (control high) for Kyn, and at 20 (control low) and 200 μM (control high) for Phe, Trp, and Tyr.

**Stability** of the analytes was acquired through processed sample stability and freeze/thaw stability in the albumin blank matrix. The stability of the processed samples in the autosampler was assessed after a minimum of 10 h, which represents a period that corresponds to the regular time of an analytical batch. All analytes were demonstrated to be stable, as no significant negative trend in the regression between the different times of injection and no decrease in peak area higher than 15% were observed (Supplementary File 1, Table S1).

Freeze/thaw stability was performed by 3 cycles and showed average results of control samples at low and high concentrations within 100 and 103% of the corresponding control samples, which proves the freeze/thaw stability of all analytes (Supplementary File 1, Table S2).

For evaluation of the precision of the analysis within a laboratory as well as the reproducibility of the method, QC samples were measured within the same laboratory but on a different instrument and prepared by a different operator, and in a different laboratory with different equipment. Although the reproducibility cannot be calculated by the experimental design used in this work, Table 4 compares the measured analyte concentrations in QC samples with different instruments prepared by different operators and measured in different laboratories and Figure 3 shows a correlation of serum samples from a set of 43 donors. All values appeared to be ± 15% compared to the control measurements (Instrument 1). In addition, real samples of healthy donors were measured in two different laboratories using the same method.

**Table 4:** Comparison of Measured Analyte Concentrations (in μM) in QC Samples^a^

analyte	matrix	QC concemtration (μM)	instrument 1	instrument 2	different operator	external laboratory 1
Kyn	albumin	1.5	1.44	1.49	1.38	1.25
		15	14.69	14.06	13.73	15.48
Trp	albumin	15	14.91	14.88	14.15	14.97
		150	150.69	149.35	140.38	150.07
Tyr	albumin	15	14.69	13.67	13.82	14.19
		150	146.86	140.65	136.78	144.27
Phe	albumin	15	14.66	14.11	13.86	14.13
		150	149.25	142.74	138.04	146.37

a QC low represents 1.5 μM for Kyn and 15 μM for Trp, Tyr, and Phe, and QC high represents 15 μM for Kyn and 150 μM for Trp, Tyr, and Phe. Samples that were prepared by a different operator were measured on instrument 2. All samples were measured in duplicate; concentrations measured on instrument 1 are the mean of three independent measurements.

**Figure 3:**
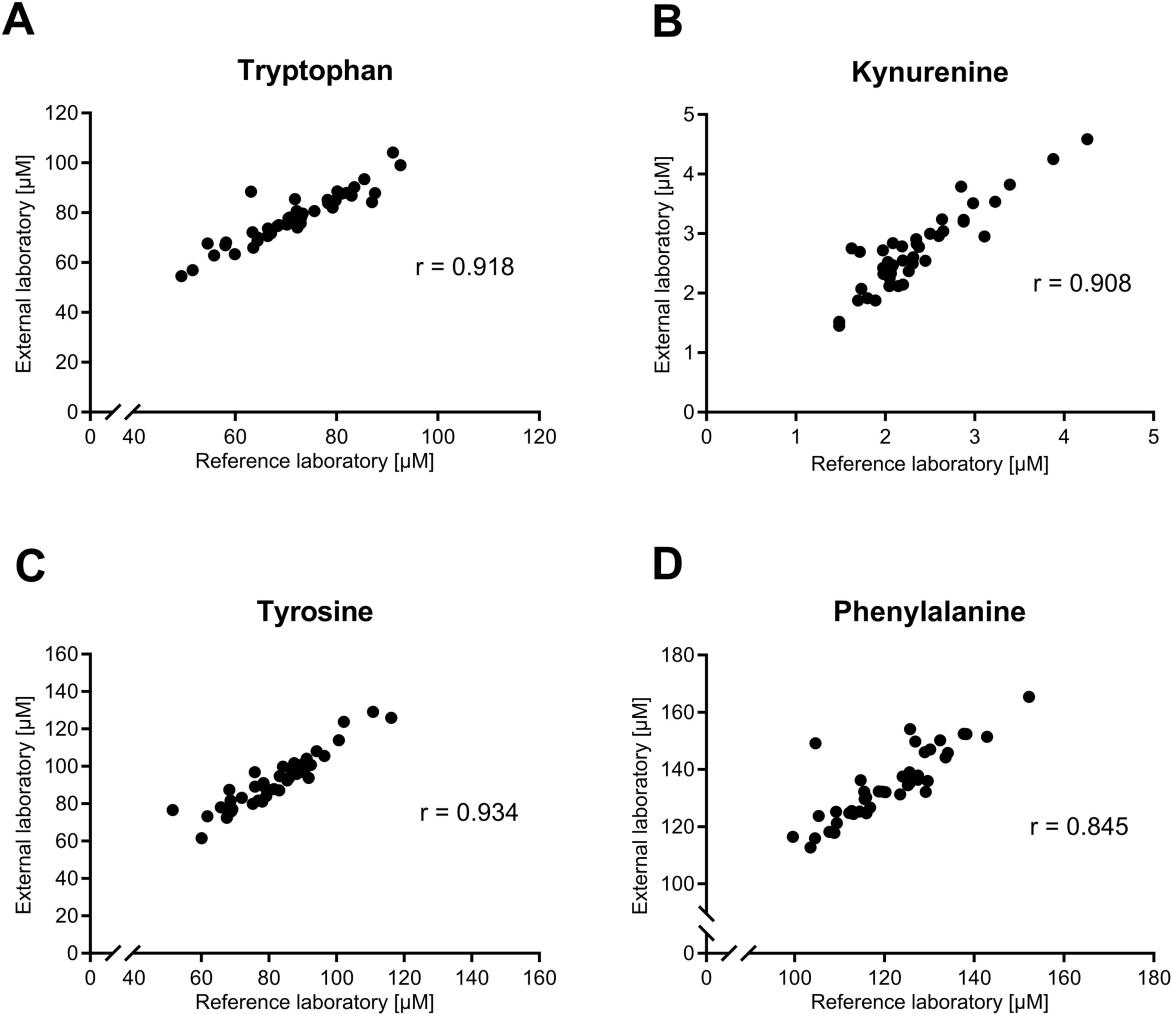
Comparison of AA concentrations Trp (A), Kyn (B), Tyr (C), and Phe (D) in 43 serum samples measured in the reference laboratory (instrument 1) and the external laboratory (external laboratory 1) with the HPLC-based method on RP18 and using UV/FLD detection (*n* = 43) (Supporting Information File 2). The Pearson correlation coefficient (*r*) indicates a strong positive linear relationship.

In order to have a further comparison with a method that is not based on the detection of native fluorescence, calibrators and standards were analyzed using the HPLC method based on ion exchange and ninhydrin-derivatization (External laboratory 2). Kyn could not be detected with this method and the Trp peak was partially overlaid by the histidine peak. However, less than a 15% difference was estimated comparing the concentrations revealed by measurements of the calibrators with the calculated concentrations of 100 μM Phe and Tyr. The correlations of Phe and Tyr concentrations of five serum samples are shown in Figure 4.

**Figure 4:**
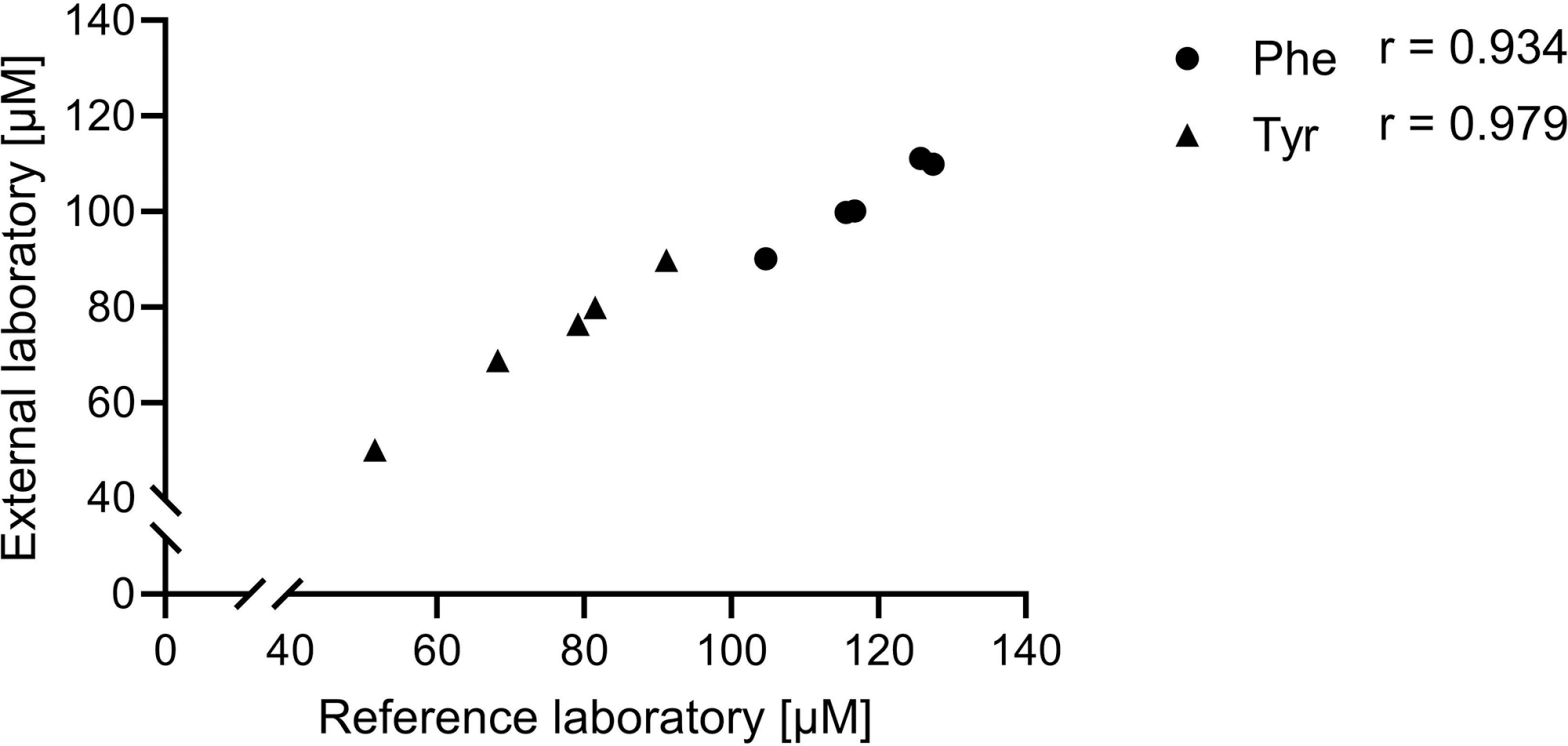
Comparison of concentration of Phe and Tyr in five serum samples measured in the reference laboratory with the HPLC-method using RP18 and UV/FLD detection and in external laboratory 2 with an HPLC-method using ion exchange and ninhydrin-derivatization (*n* = 5) (Supporting Information File 2). The Pearson correlation coefficient (*r*) indicates a strong positive linear relationship.

## Discussion

The multiplex analysis of endogenous metabolites in human serum or plasma is inherently challenging due to matrix variations and varying metabolite concentrations. In this study, we report a simple, rapid, and accurate method for the simultaneous quantification of Kyn, Phe, Trp, and Tyr in human plasma and serum. This approach offers several advantages, including the need of only a small sample volume, a short analysis time, no derivatization steps, minimal sample preparation, a simple analytical procedure, and lower equipment costs compared to LC-MS approaches. AAs are quantified based on their absorbance (Kyn) and natural fluorescence (Tyr, Phe, Trp) using a DAD-equipped HPLC in combination with a fluorescence detector, eliminating the need for sample derivatization and increasing sensitivity. The method is based on previously reported HPLC techniques for quantifying Trp, Kyn, Phe, and Tyr in human plasma and serum and uses albumin-based calibrators.^29,31,34,37^

The calibration range was chosen to match the expected analyte concentrations in real samples. Geisler et al. reported 53.1 μM Trp, 2.48 μM Kyn, 59.5 μM Tyr and 83.4 μM Phe as the 95th percentile of healthy individuals.^38^ Significant gender differences were reported for Trp and Tyr concentrations, and as a trend for Phe.^38^ Moreover, it is well established that both age and immune status affect the blood concentration of AAs.

The validation results demonstrated that the method presented is selective, exhibits excellent linearity over a wide calibration range, and minimal bias, and has high accuracy in both albumin and charcoal-depleted plasma/serum matrices. The calculated 95% tolerance intervals meet the acceptance criteria, further confirming the reliability of the method.

In addition, stability analyses showed that the analytes remained stable during storage of the processed samples and freeze/thaw cycles. Interlaboratory comparisons confirmed the consistency and reproducibility of the method between different instruments and operators, increasing its applicability in different research environments. The correlation of serum samples measured in different laboratories obtained correlation coefficients of the analytes higher than 0.84, indicating an acceptable reproducibility of the method with different equipment in different laboratories.

Recent work has advanced methods for analysis of AAs with simplified preanalytics across a range of detection platforms (Supplementary File 1, Table S3). Chromatographic approaches, such as HPLC–UV/FLD, including the method presented here, remain attractive for serum and plasma because they are straightforward, require only small sample volumes, and avoid derivatization, while still providing robust sensitivity (Table 2) and reproducibility. In contrast, previously reported HPLC methods rely on labor-intensive derivatization steps (e.g., ninhydrin or phenylisothiocyanate) to achieve sufficient sensitivity, which increases analytical complexity and potential sources of error.^24^ Our workflow eliminates this need, while demonstrating comparable or superior sensitivity and precision with minimal sample preparation. MS-based methods offer excellent selectivity and ultralow detection limits, but require costly instrumentation, specialized expertise, and often complex protocols, which may limit their routine clinical use.^24,39–45^

Alternative detection methods based on the use of conducting polymers have recently emerged for small molecule analysis, offering promising new avenues beyond conventional UV, fluorescence, and MS detection; however, these approaches are not yet sufficiently established or validated for quantitative applications in clinical chemistry.^46,47^

Thus, our method provides a balanced solution: a robust, derivatization-free HPLC–UV/FLD workflow that is easy to implement, cost-effective, and well-suited for targeted AA analysis in human blood samples. Importantly, to our knowledge, this is the first HPLC–UV/FLD assay for Trp, Kyn, Phe, and Tyr in both plasma and serum that has been explicitly validated according to GTFCH guidelines, further underlining its suitability for clinical application.

In conclusion, the validated HPLC method provides a robust and efficient technique for the quantification of Trp, Kyn, Phe, and Tyr in serum and plasma samples. Its precision, accuracy and selectivity make this method a valuable tool for researchers investigating the role of these AAs in a variety of health and disease contexts, and it offers the possibility of easy implementation in research, routine diagnostics and personalized medicine.

## Supplementary Material





## Data Availability

Data are available in the Supplementary File 1 and File 2.

## Notes

Figures S1–S4; Tables S1–S3 (PDF)
Data for calibration of the analytes in charcoal-depleted serum; data for calibration of the analytes in charcoal-depleted plasma; data for calibration of the analytes in albumin; data for Table 2; data for accuracy; data for Table 3; data for Table 4; processed sample stability (Table S1); freeze thaw stability (Table S2); interlaboratory comparison of Figures 3 and 4; recovery data in % for Table 3; extraction efficiency for Table 3 (XLSX)
