# Supplementary material for: A Validated Method for the Simultaneous Measurement of Tryptophan, Kynurenine, Phenylalanine, and Tyrosine by High-Performance Liquid Chromatography–Ultraviolet/Fluorescence Detection in Human Plasma and Serum
Source: ACS Omega. 2026 Jan 16;11(4):5201–10. doi: 10.1021/acsomega.5c07457 (PMC12878734; doi:10.1021/acsomega.5c07457)
Supplement: Supplementary file 1 [file ao5c07457_si_001.pdf]

A validated method for the simultaneous measurement of tryptophan, kynurenine, phenylalanine and tyrosine by HPLC-UV/FLD in human plasma and serum

Lucia Parráková[1+], Cornelia A. Karg[1+], Stefanie Hofer[1], Pablo Monfort-Lanzas[1,2], Celina Wilgermein[3], Kevin Allmer[4], Sabine Scholl-Bürgi[5], Anita Siller[6], Harald Schennach[6], Simon Geisler[1], Dietmar Fuchs[1,7], Thomas K. Felder[4,8], Johanna M. Gostner[1,9]*

[1] Institute of Medical Biochemistry, Biocenter, Medical University of Innsbruck, 6020 Innsbruck, Austria;

[2] Institute of Bioinformatics, Biocenter, Medical University of Innsbruck, 6020 Innsbruck, Austria;

[3] Division of Psychiatry I, Department of Psychiatry, Psychotherapy, Psychosomatics and Medical Psychology, Medical University of Innsbruck, 6020 Innsbruck, Austria;

[4] Department of Laboratory Medicine, Paracelsus Medical University, 5020 Salzburg, Austria;

[5] Department of Paediatrics I, Medical University of Innsbruck, 6020 Innsbruck, Austria;

[6] Central Institute for Blood Transfusion and Immunology, University Hospital Innsbruck, Tirol Kliniken GmbH, 6020 Innsbruck, Austria;

[7] Institute of Biological Chemistry, Medical University of Innsbruck, 6020 Innsbruck, Austria;

[8] Institute of Pharmacy, Paracelsus Medical University, 5020 Salzburg, Austria;

[9] Core Facility Metabolomics II, Biocenter, Medical University of Innsbruck, 6020 Innsbruck, Austria

(+) Lucia Parráková and Cornelia A. Karg contributed equally to this work.

(*) Corresponding author:
Johanna M. Gostner
johanna.gostner@i-med.ac.at

Supplementary Information

Figure S1. UV-Vis absorption spectra of analytes measured in aqueous solution (200 µM). Spectra were recorded on a NanoDrop One^{c} spectrophotometer (Thermo Fisher Scientific, USA) using a quartz cuvette over the wavelength range of 180 – 400 nm. Ultrapure water served as the blank for baseline correction. All measurements were performed at room temperature.

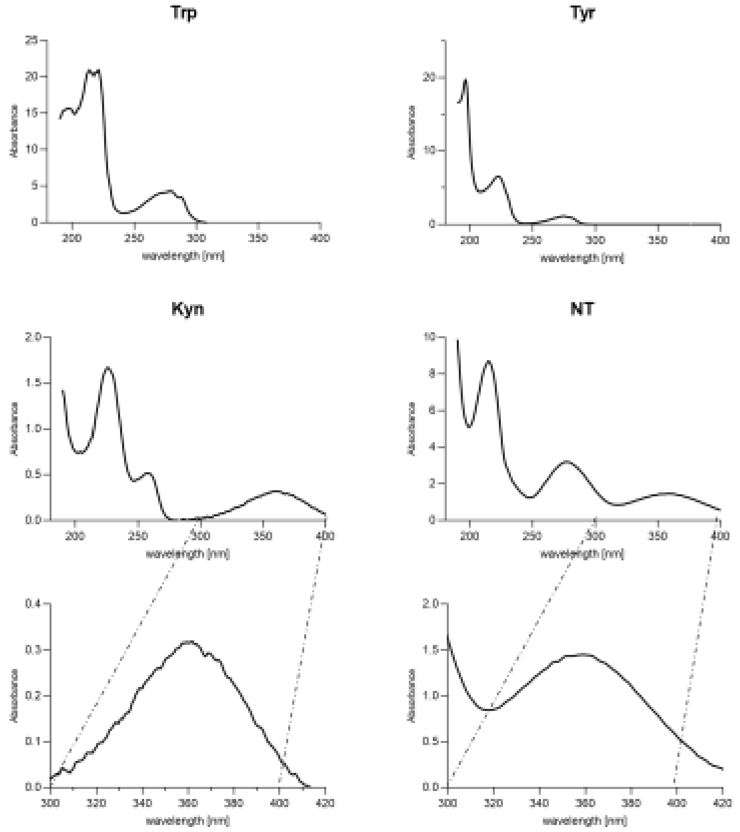

Figure S2: Fluorescence emission spectra of analytes were recorded in aqueous solution at a concentration of 66 µM using a Cary Eclipse fluorescence spectrometer equipped with a 1 cm quartz cuvette. Emission spectra were acquired over the 280–500 nm range at the excitation wavelengths indicated in the figures, with excitation and emission slit widths set to 5 nm. All measurements were performed at room temperature. Kyn and NT were measured at additional excitation wavelengths to illustrate their weak fluorescence at the monitored emission wavelengths and to demonstrate their suitability for detection by UV absorption rather than fluorescence.

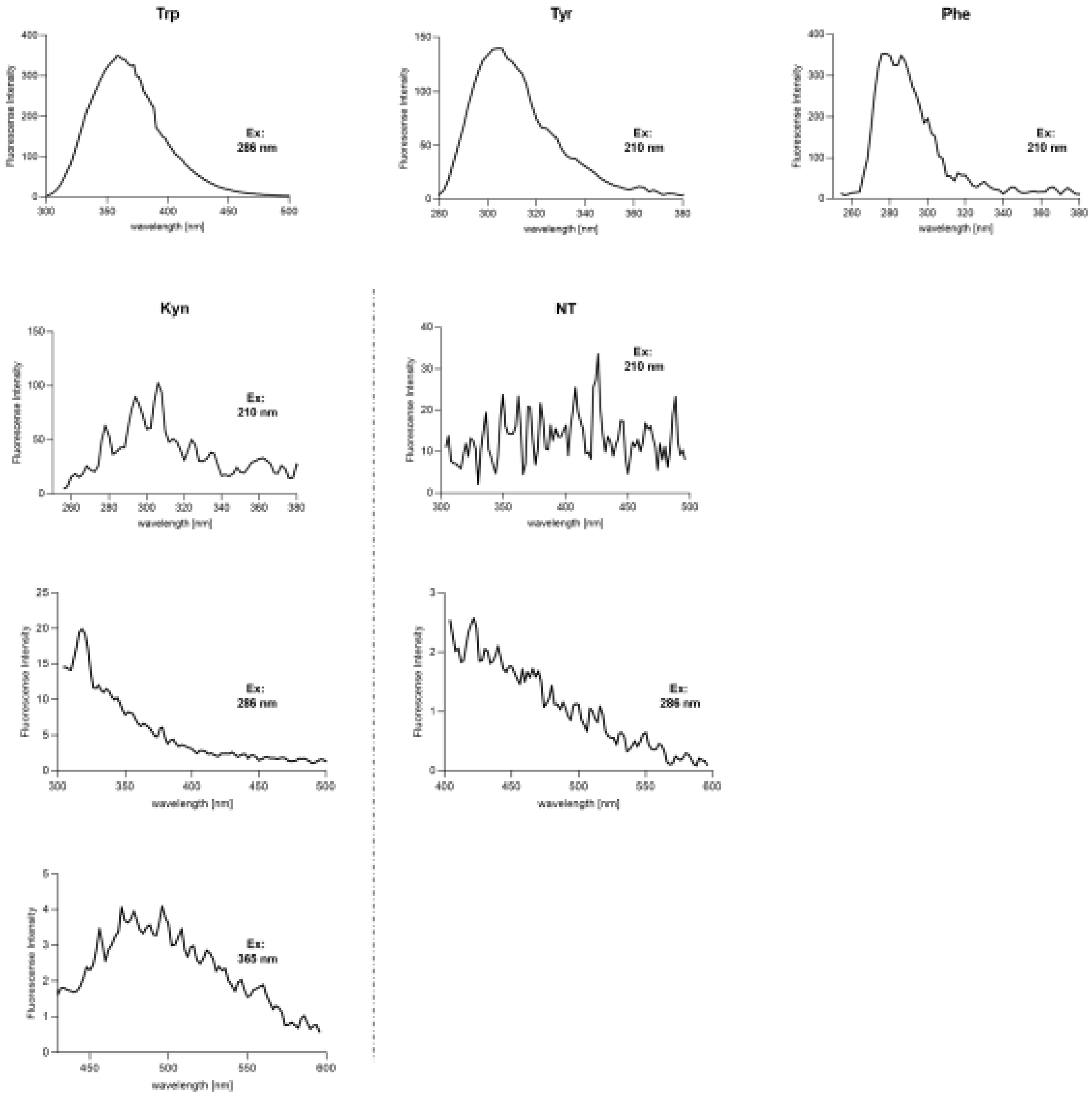

Figure S3: Chromatograms of the albumin (A), charcoal-depleted serum (CDS) (B), and charcoal-depleted plasma (CDP) (B) blanks containing the internal standard nitrotyrosine (NT).

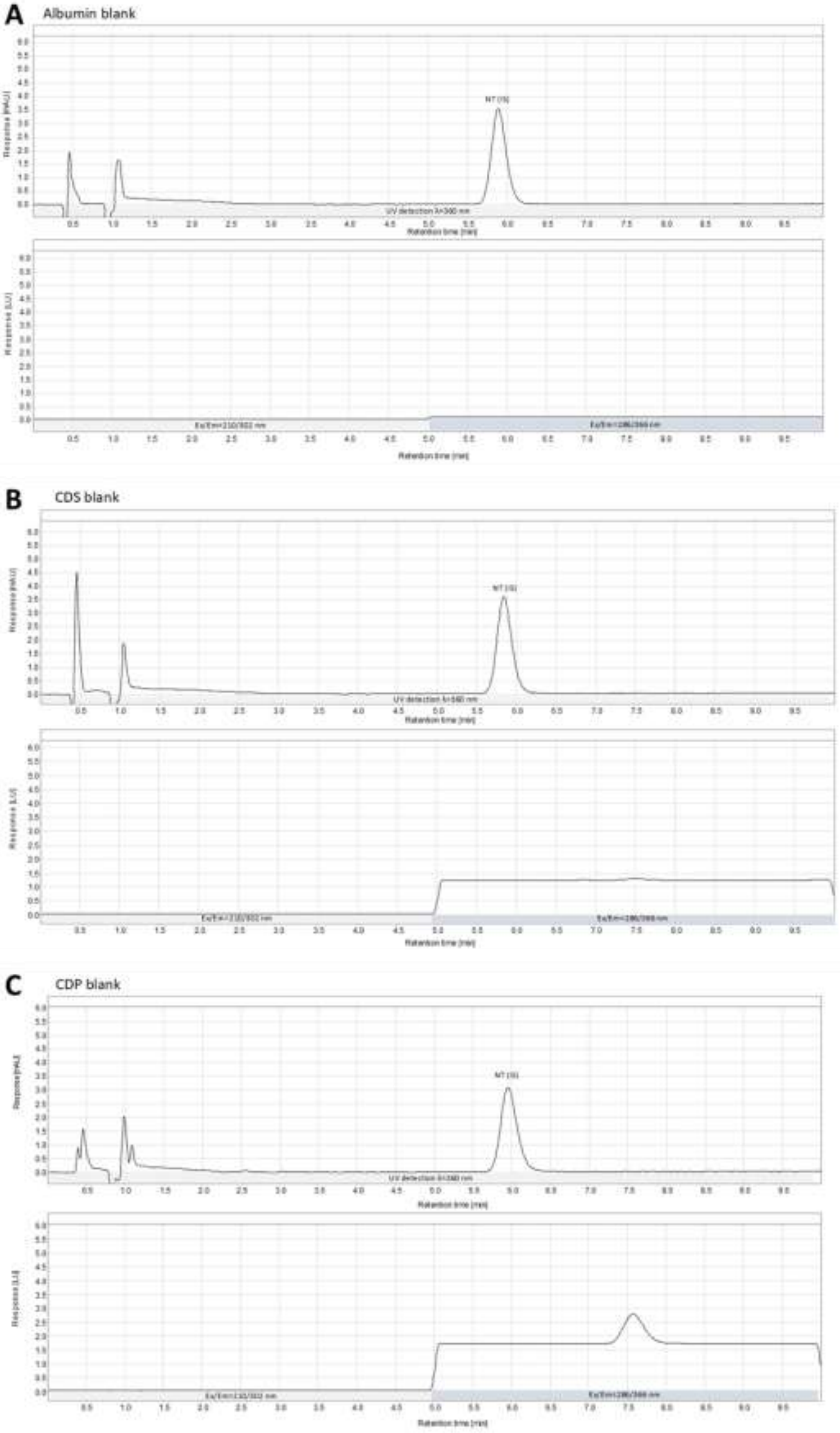

Figure S4: Formulas used for the calculations according to guidelines of the Gesellschaft für Toxikologische und Forensische Chemie (GTFCH) (1).

Repeatability

Calculation of the repeatability variance

$$S_r^2 = \frac{\sum_{i=1}^{p} \sum_{k=1}^{n} (x_{ik} - \underline{x_i})^2}{p(n-1)}$$

S^2r – Repeatability variance

p – number of days

n – number of repetitions per day

x_{ik} – measurement *k* on day *i*

$\underline{x_i}$ – average of the n measurements on day *i*

Calculation of repeatability from repeatability variance

$$RSDr\ (\%) = \frac{\sqrt{S_r^2}}{\underline{x}} \cdot 100$$

RSDr – Repeatability (precision)

S^2r – Repeatability variance

$\underline{X}$ – Average of all measurements

Time-different intermediate precision

Calculation of time-different intermediate precision

$$S_t^2 = \frac{\sum_{i=1}^{p} (\underline{x}_{ik} - \underline{x})^2}{p-1} - \frac{S_r^2}{n}$$

S_t^2 – variance between days

$\underline{x_i}$ – average of the n measurements on day i

$\underline{X}$ – average of all measurements

p – number of days

S_r^2 – repeatability variance

n – number of repetitions per day

Calculation of time-different intermediate precision

$$RSD(T)\ (\%) = \frac{\sqrt{S_t^2 + S_r^2}}{\underline{x}} \cdot 100$$

RSD(T) – between days inter-laboratory precision

S_t^2 – variance between days

S_r^2 – repeatability variance

$\underline{X}$ – average of all measurements

Calculation of the 95% ß-tolerance interval

Bias

$$Bias\ [\%] = \frac{\underline{x} - \mu}{\mu} \cdot 100$$

$\underline{x}$ – average of all measurement

µ - accepted reference values

95% β-tolerance interval

$R = \frac{S_t^2}{S_r^2}$

$$B = \sqrt{\frac{R+1}{n \cdot R + 1}}$$

$$f = \frac{(R+1)^2}{\left(R + \left(\frac{1}{n}\right)\right)^2 / (p-1) + (1 - \left(\frac{1}{n}\right))/pn}$$

$$L_U[\%] = Bias[\%] - t_{f:0.975} \cdot \sqrt{1 + \frac{1}{p \cdot n \cdot B^2}} \cdot RSD_{(T)}[\%]$$

$$L_O[\%] = Bias[\%] + t_{f:0.975} \cdot \sqrt{1 + \frac{1}{p \cdot n \cdot B^2}} \cdot RSD_{(T)}[\%]$$

Lu [%] – lower limit of the 95% β-tolerance interval

Lo [%] – Upper limit of the 95% β-tolerance interval

f – degrees of freedom

tf;0,975 – 97,5% percentile of the t-distribution with f degrees of freedom

RSD(T) – between days inter-laboratory precision

p – number of days

n – number of repetitive measurements per day

S_t^2 – variance between days

S_r^2 – repeatability variance

B – Adjusts for days and replicates in precision estimation

Table S1. Processed sample stability assessed by comparing control samples at low and high concentrations (2 and 15 µM for Kyn, 20 and 150 µM for Trp, Tyr and Phe) during a regular time of an analytical batch. Values show ratio of the samples after 11 hours compared to control samples at the beginning of the measurement in %.

	**Peak area ratio of timepoint 660 to 0 in %**			
**Concentration of control samples (µM)**	**Kyn**	**Phe**	**Trp**	**Tyr**
20 (2 for Kyn)	98.5	106.3	103.9	103.3
150 (15 for Kyn)	101.2	106.0	104.4	104.4

Table S2. Freeze/thaw stability of control samples at low and high concentrations (0.2 and 15 µM for Kyn, 2 and 150 µM for Trp, Tyr and Phe). Values show ratio of the samples after 3 freeze/thaw cycles compared to control samples, which were only thawed once, in %.

	**Peak area ratio of samples after 3 freeze cylces in comparison to ctrl in %**			
**Concentration of control samples (µM)**	**Kyn**	**Phe**	**Trp**	**Tyr**
2 (0.2 for Kyn)	101.4	102.6	100.3	100.2
150 (15 for Kyn)	100.5	100.1	99.7	100.0

Table S3: Comparative overview on selected chromatographic and mass spectrometric methods for amino acid analysis. The table includes both the methods on which the current work is based and more recent methodological developments. Sensitivities are reported in either mass-per-volume units or molar concentration units, as used in the original publications (bold), and were recalculated accordingly for comparison (* µmol/kg was recalculated to µM with 1 kg = 1l). (FLD – fluorescence detection, (L)LOD – (lower) limit of detection, (L)LOQ – (lower) limit of quantification, (U)HPLC – (ultra)-high-performance liquid chromatography, GTFCH – Gesellschaft für toxikologische und forensische Chemie, HILIC – hydrophilic interaction liquid chromatography, ICH – International Council for Harmonisation, Kyn – kynurenine, LC – liquid chromatography, MS – mass spectrometry, Phe – phenylalanine, PITC – phenylisothiocyanate, Trp – tryptophan, Tyr – tyrosine, UV – ultraviolet detection)

Study	Method	Sensitivity [µM]	Sensitivity [mg/l]	Matrix	Derivatization	Comments	Reference
**HPLC-UV and/or FLD based methods**							
Parrakova and Karg et al. (this study)	HPLC-UV/FLD	**LOD / LLOQ<br>Phe 0.39 / 1.56<br>Tyr 0.08 / 0.78<br>Trp 0.19 / 0.78<br>Kyn 0.31 / 0.63<br>(plasma)**	**LOD / LLOQ<br>Phe 0.064 / 0.26<br>Tyr 0.014 / 0.14<br>Trp 0.039 / 0.16<br>Kyn 0.065 / 0.13<br>(plasma)**	Plasma, serum	No	GTFCH-validated	(1)
Eid et al. (2022)	HPLC-UV	LOD / LOQ<br>Phe 42.4 / 133.2<br>Tyr 55.2 / 165.6<br>Trp 19.6 / 24.5	**LOD / LOQ<br>Phe 7 / 22<br>Tyr 10 / 30<br>Trp 4 / 5**	Dietary supplements	No	ICH-validated	(2)
Laich et al. (2002)	HPLC-UV/FLD	N.A.	N.A.	Serum	No		(3)
Michel et al. (2020)	HPLC-ninhydrin-photometry	**LOD / LLOQ<br>Phe 3.3 / 6.6<br>Tyr 1.1 / 2.2<br>Trp 14.8 / 29.5**	LOD / LLOQ<br>Phe 0.55 / 0.26<br>Tyr 0.20 / 0.40<br>Trp 3.02 / 6.02	Serum	Yes	Ninhydrin derivatization-based	(4)
Neurauter et al. (2013)	HPLC-FLD	**LOD<br>Phe 0.31<br>Tyr 0.31**	LOD<br>Phe 0.05<br>Tyr 0.06	Serum	No		(5)
Widner et al. (1999)	HPLC-UV/FLD	N.A.	N.A.	Serum	No	Linearity was tested between 0.09 and 9.84 µM for Kyn and 0.06 and 220 µM for Trp.	(6)
**LC-MS/MS based methods**							
Elif Öztürk Er et al. (2021)*	LC-MS/MS	LOD / LOQ<br>Phe 0.05 / 0.16<br>Tyr 0.07 / 0.23	LOD / LOQ<br>Phe 0.008 / 0.026<br>Tyr 0.013 / 0.042	Plasma, urine	No	HILIC column; quadruple isotope dilution mass spectrometry	(7)
Kaliszewska et al. (2025)	LC–MS	LOD-ranges / LOQ-ranges<br>Phe 0.006 – 1.01 / 0.152 – 18.2<br>Tyr 0.0055 – 0.916 / 0.138 – 16.6	**LOD-ranges<br>0.001–0.166<br>LOQ ranges**	Plasma, cerebrospinal fluid	No		(8)

		Trp 0.0049 – 0.813 / 0.122 – 14.7	**0.025–3**				
Lin et al. (2025)	LC-MS/MS	LLOQ<br>Phe 0.61<br>Tyr 0.55<br>Trp 0.49	**LLOQ**<br>**Phe 0.1**<br>**Tyr 0.1**<br>**Trp 0.1**	Plasma	No	HILIC column	(9)
Michel et al. (2020)	UHPLC-PITC-MS/MS	**LOD / LLOQ**<br>**Phe 0.1 / 5.0**<br>**Tyr 0.5 / 5.5**<br>**Trp 0.5 / 5.0**	LOD / LLOQ<br>Phe 0.02 / 0.83<br>Tyr 0.09 / 0.91<br>Trp 0.10 / 1.02	Serum	Yes	Phenylisothiocyanate derivatization	(4)
Németh et al. (2023)	LC-MS/MS	**LLOD / LLOQ**<br>**Phe 0.0005 / 0.001**<br>**Tyr 0.001 / 0.005**<br>**Trp 0.0001 / 0.0005**	LLOD / LLOQ<br>Phe 0.00008 / 0.00017<br>Tyr 0.00018 / 0.00091<br>Trp 0.00002 / 0.0001	Serum	No	Normal-phase HPLC column (silica stationary phase, reversed-phase eluents)	(10)
Ortega et al. (2024)	LC/MS	LOD / LOQ<br>Phe 0.003 / 0.009<br>Tyr 0.050 / 0.166<br>Trp 0.012 / 0.040	**LOD / LOQ**<br>**Phe 0.00046 / 0.00152**<br>**Tyr 0.009 / 0.03**<br>**Trp 0.00247 / 0.00823**	Milk, tissue, feed and soy flour	No	Z-HILIC column	(11)
Wang et al. (2024)	UHPLC-MS/MS	LOD / LOQ<br>Phe 0.0245 / 0.0733<br>Tyr 0.663 / 1.99<br>Trp 0.0411 / 0.123	**LOD / LOQ**<br>**Phe 0.004 / 0.0121**<br>**Tyr 0.12 / 0.3595**<br>**Trp 0.0084 / 0.0252**	Meat	No		(12)
Yin et al. (2024)	LC-MS/MS	**LOD / LLOQ**<br>**Phe 0.33 / 0.65**<br>**Tyr 1.95 / 3.91**<br>**Trp 1.95 / 3.90**<br>**Kyn 0.87 / 1.73**	LOD / LLOQ<br>Phe 0.05 / 0.11<br>Tyr 0.35 / 0.71<br>Trp 0.40 / 0.80<br>Kyn 0.18 / 0.36	Plasma	No	HILIC column	(13)

References:

1. Peters, F. T.; Hartung, M.; Herbold, M.; Schmitt, G.; Daldrup, T.; Mußhoff, F. Anforderungen an die Validierung von Analysenmethoden. Toxichem Krimtech 2009, 76 (3), 185–208.

2. Eid SM, Farag MA, Bawazeer S. Underivatized Amino Acid Chromatographic Separation: Optimized Conditions for HPLC-UV Simultaneous Quantification of Isoleucine, Leucine, Lysine, Threonine, Histidine, Valine, Methionine, Phenylalanine, Tryptophan, and Tyrosine in Dietary Supplements. ACS Omega. 2022;7(35):31106-14.

3. Laich A, Neurauter G, Widner B, Fuchs D. More rapid method for simultaneous measurement of tryptophan and kynurenine by HPLC. Clin Chem. 2002;48(3):579-81.

4. Michel M, Salvador C, Wiedemair V, Adam MG, Laser KT, Dubowy KO, et al. Method comparison of HPLC-ninhydrin-photometry and UHPLC-PITC-tandem mass spectrometry for serum amino acid analyses in patients with complex congenital heart disease and controls. Metabolomics. 2020;16(12):128.

5. Neurauter G, Scholl-Burgi S, Haara A, Geisler S, Mayersbach P, Schennach H, et al. Simultaneous measurement of phenylalanine and tyrosine by high performance liquid chromatography (HPLC) with fluorescence detection. Clin Biochem. 2013;46(18):1848-51.

6. Widner B, Werner ER, Schennach H, Fuchs D. An HPLC method to determine tryptophan and kynurenine in serum simultaneously. Adv Exp Med Biol. 1999;467:827-32.

7. Öztürk Er E, Özbek B, Bakırdere S. Determination of seventeen free amino acids in human urine and plasma samples using quadruple isotope dilution mass spectrometry combined with hydrophilic interaction liquid chromatography – Tandem mass spectrometry. Journal of Chromatography A. 2021;1641:461970.

8. Kaliszewska A, Struczynski P, Baczek T, Niedzwiecki M, Konieczna L. Comprehensive Analysis and Comparison of Amino Acid Levels in Cerebrospinal Fluid and Plasma of Children with Leukemia by the LC-MS Technique. Int J Mol Sci. 2025;26(5).

9. Lin J, Song Y, Zhang Y, Ke T, Ou F, Zeng K, et al. A reliable LC-MS/MS method for the quantification of natural amino acids in human plasma and its application in clinic. J Pharm Biomed Anal. 2025;256:116672.

10. Nemeth K, Szatmari I, Tokesi V, Szabo PT. Application of Normal-Phase Silica Column in Hydrophilic Interaction Liquid Chromatography Mode for Simultaneous Determination of Underivatized Amino Acids from Human Serum Samples via Liquid Chromatography-Tandem Mass Spectrometry. Curr Issues Mol Biol. 2023;45(12):9354-67.

11. Ortega AF, Zhao H, Van Amburgh ME. Development and Validation of a Method for Hydrolysis and Analysis of Amino Acids in Ruminant Feeds, Tissue, and Milk Using Isotope Dilution Z-HILIC Coupled with Electrospray Ionization Triple Quadrupole LC-MS/MS. J Agric Food Chem. 2024;72(1):833-44.

12. Wang M, Guo J, Lin H, Zou D, Zhu J, Yang Z, et al. UHPLC-QQQ-MS/MS method for the simultaneous quantification of 18 amino acids in various meats. Front Nutr. 2024;11:1467149.

13. Yin X, Baldoni I, Adams E, Van Schepdael A. Analysis Profiling of 48 Endogenous Amino Acids and Related Compounds in Human Plasma Using Hydrophilic Interaction Liquid Chromatography-Tandem Mass Spectrometry. Molecules. 2024;29(24).
